# PD-1 combined with lenvatinib and TACE for the transformational treatment of hepatocellular carcinoma combined with portal vein tumor thrombus: a case report and literature review

**DOI:** 10.3389/fonc.2023.1199143

**Published:** 2023-10-05

**Authors:** Sheng Liu, Rui Xiong, Chuanyi Duan, Jiang Tang, Tao Yin, Sisi Dai

**Affiliations:** ^1^ Department of Hepatobiliary Surgery, Hubei Cancer Hospital, Tongji Medical College, Huazhong University of Science and Technology, Wuhan, Hubei, China; ^2^ Department of Anaesthesiology, Xiangya Hospital, Central South University, Changsha, Hunan, China

**Keywords:** PD-1, lenvatinib, hepatocellular carcinoma, portal vein tumor thrombus, TACE

## Abstract

**Background:**

The prognosis of hepatocellular carcinoma combined with portal vein tumor thrombus is poor, with a median survival of only 3-6 months. PD-1 combined with targeted therapy may provide an opportunity for patients with BCLC C stage hepatocellular carcinoma combined with portal vein tumor thrombus to undergo radical surgery, significantly prolonging their survival time.

**Case presentation:**

A middle-aged 51-year-old male who was diagnosed with hepatocellular carcinoma combined with portal vein main stem tumor thrombus at our center in May 2020, with a BCLC stage of C, liver cirrhosis, HBV infection, and preoperative evaluation as unresectable. The liver function was Child-Pugh A. The initial treatment was lenvatinib combined with PD-1 therapy, followed by one cycle of TACE treatment. The tumor and thrombus volume significantly reduced, followed by continuous TACE combined with immunotherapy and targeted therapy, leading to the appearance of portal vein main stem emboli. After multidisciplinary discussion, surgical resection was performed, and the embolus was removed, achieving a cure. The patient has been tumor-free for over 34 months.

**Conclusion:**

PD-1 combined with lenvatinib and local TACE create conditions for radical surgery, and it is hoped that more real-world research data can provide better evidence for the transformational treatment of hepatocellular carcinoma combined with portal vein tumor thrombus.

## Introduction

Primary liver cancer is the sixth most common cancer globally, with approximately 906,000 new cases and 830,000 deaths (1). In China, hepatocellular carcinoma (HCC) is the fourth leading cause of cancer-related deaths (2). Research has shown that only 30-40% of patients can undergo surgical treatment after their first visit, and most patients are diagnosed at a late stage, where treatment with chemotherapy, ablation therapy, and transarterial chemoembolization (TACE) is limited. Therefore, treating late-stage liver cancer is difficult, but targeted drugs such as sorafenib and lenvatinib have achieved good treatment results in recent years. In 2018, lenvatinib was recommended as a first-line targeted therapy for advanced liver cancer by NCCN guidelines (3). It can inhibit multiple intracellular and cell surface kinases, providing a dual mechanism of action. On the one hand, it directly inhibits tumor growth by blocking some signal transduction pathways; on the other hand, it can prevent neovascularization, making it one of the most widely used anti-tumor-targeted drugs (4, 5).

It has been shown that TACE combined with targeted drugs such as lenvatinib can significantly improve patients’ OS and PFS (6). TACE blocks the nutrient vessels of the tumor through interventional means and injects chemotherapy drugs, significantly improving the efficiency of embolization (7). It has achieved satisfactory results in clinical trials and is recommended for BCLC B-stage liver cancer patients (8). In addition, the 2020 Asia-Pacific Primary Liver Cancer Expert Consensus also unanimously recognized that TACE plays an important auxiliary role in the treatment of mid-to-late-stage liver cancer and can improve treatment efficacy (8). The progress in TACE technology, especially the refinement of exceptionally stable and uniform iodized oil formulations (9), has yielded encouraging outcomes in the management of liver cancer at intermediate to advanced stages. This progress has brought about a profound transformation in the paradigm of TACE therapy for liver cancer (10). Furthermore, the integration of TACE and immunotherapy represents a burgeoning therapeutic paradigm in the context of liver cancer (11). This approach endeavors to enhance treatment outcomes by synergizing the localized tumor-targeting properties of TACE with the systemic immune-enhancing attributes of immunotherapy (12). TACE can induce an immunogenic microenvironment within the tumor, rendering it more vulnerable to immune-mediated assaults. Concurrently, immunotherapy bolsters the body’s immune responses against cancerous cells, encompassing those subjected to TACE (13).

Immune checkpoint inhibitors (ICIs), including PD-1 and CTLA-4, are also widely used in clinical practice. They have been approved as second-line treatments for HCC patients previously treated with sorafenib (14, 15). Immune therapy has shown excellent results in many clinical trials. PD-1 has shown significant anti-tumor effects as a treatment for HCC, particularly when used in combination with tyrosine kinase inhibitors (16, 17). PD-1 combined with targeted therapy has shown excellent results in patients, and clinical trials using atezolizumab combined with bevacizumab to treat unresectable HCC are highly anticipated, with direct entry into first-line treatment (18, 19). The study of PD-1 combined with lenvatinib also suggests that patients with inoperable liver cancer may benefit. A prospective study by Lu et al. showed that the successful conversion rate for immune therapy combined with lenvatinib in treating HCC with vascular invasion was 42.4% (20).

Here, we report a case of successful treatment of BCLC C-stage liver cancer using PD-1 combined with lenvatinib, among other treatments, at our center.

## Case presentation

**Figure 1:**
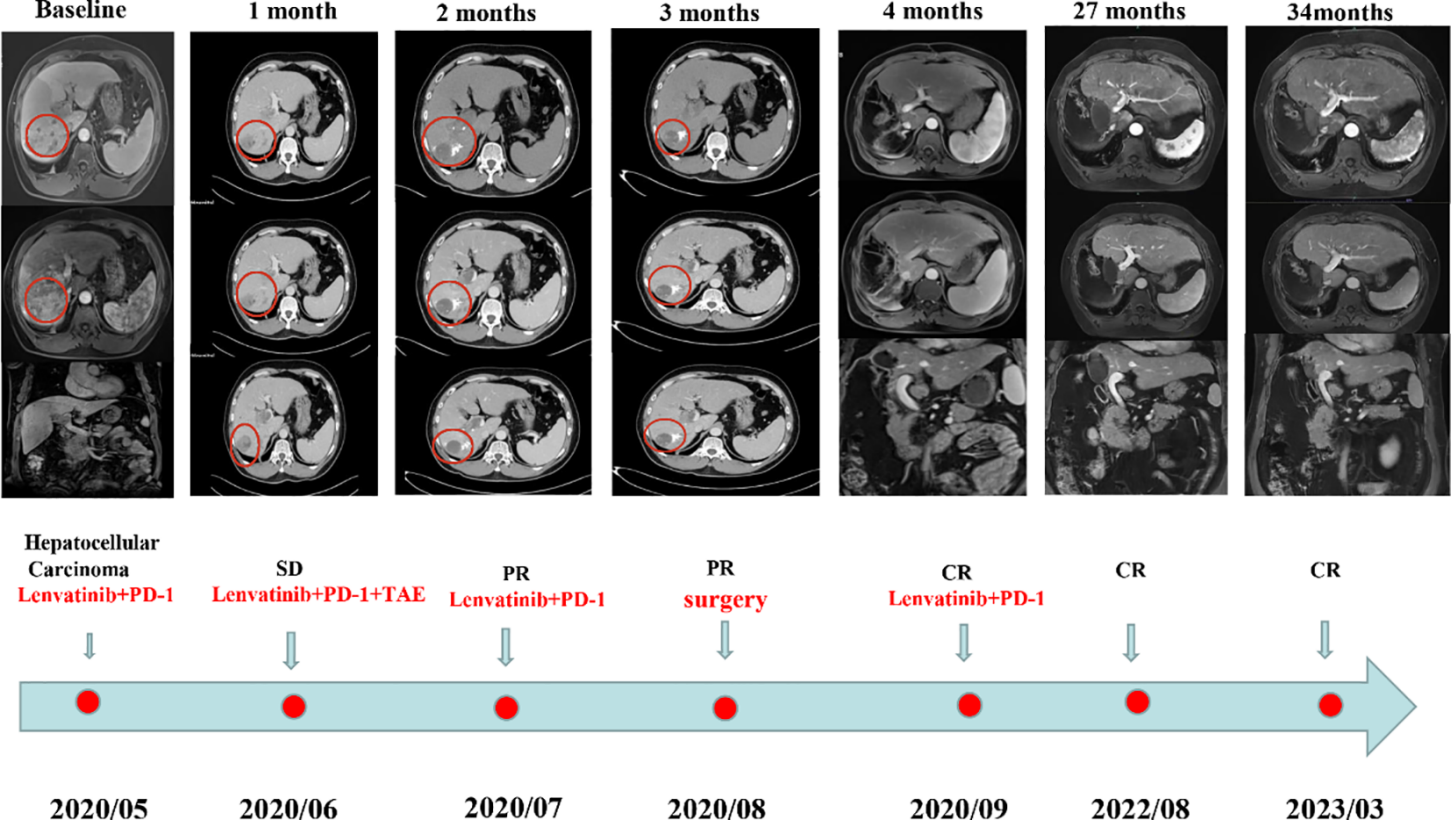
The details of the subsequent clinical course. Baseline: MRI examination revealed a lesion of approximately 1098cm in size in the right liver lobe segments S7 and S6, as well as a main portal vein thrombus.; 1 month: After three weeks of combined targeted therapy and immunotherapy, the patient’s tumor was stable according to mRECIST; 2 months: After the patient received the second round of combination targeted and immunotherapy, the response was assessed as partial response (PR); 3 months: After 4 weeks of TACE treatment, the treatment response was reassessed and again determined to be PR; 4 months: After undergoing another round of combined targeted and immunotherapy, the response was assessed as a complete response (CR); 27 months and 34 months: The patient was treated with the original regimen for six months after surgery and has been without recurrence for over 27months and 34 months.

In May 2022, a male patient, aged 50, sought medical evaluation. He had a documented history of hepatitis B infection and liver cirrhosis. His height was 169 cm, weight was 80 kg, and he belonged to Child-Pugh Liver Function Class A, indicating a state of moderate cirrhosis. MRI examination revealed a lesion of approximately 1098cm in size in the right liver lobe segments S7 and S6, as well as a main portal vein thrombus (baseline, **Figure 1**). The patient had an Eastern Cooperative Oncology Group Performance Status (ECOG-PS) of 1 and a Child-Pugh score of 5. The levels of protein induced by vitamin K absence-II (PIVKA-II) and alpha-fetoprotein (AFP) were 27603 ng/ml and 4.5 ng/ml, respectively.

**Figure 2:**
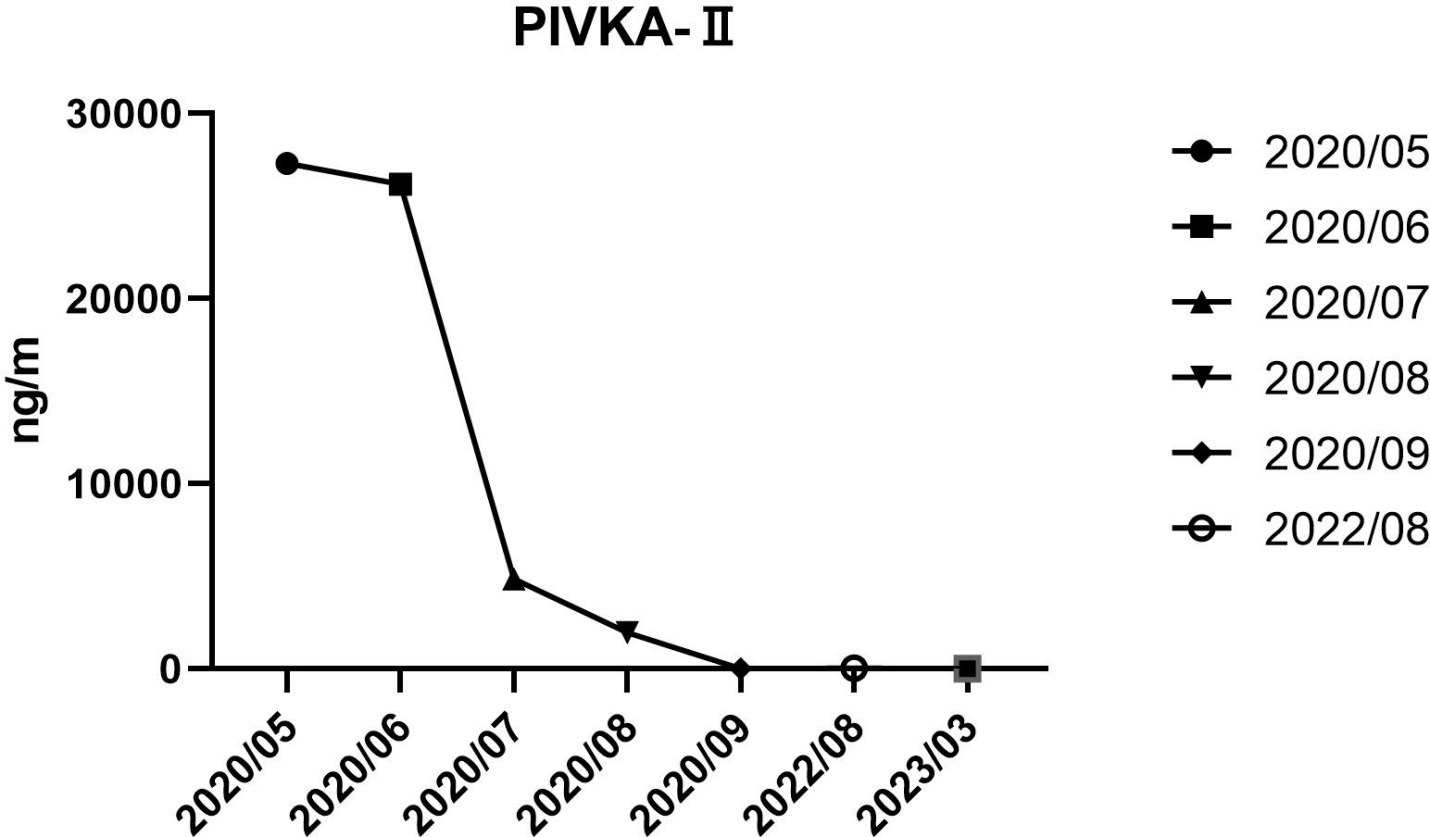
Trends in PIVKA-II level from May 2020 to August 2022.

After a multidisciplinary discussion, it was deemed that the patient was not suitable for radical surgical resection. The initial treatment plan was a one-cycle combination of PD-1 (Camrelizumab) (Jiangsu Hengrui Medicine Co. Ltd., Jiangsu, China) and lenvatinib (Eisai Co., Ltd., Tokyo, Japan). PD-1 was given at a fixed dose of 200 mg every 3 weeks intravenously, and lenvatinib was administered at a dose of 8mg/day. After three weeks, the patient’s tumor was stable according to mRECIST (**Figure 1**, 1 month) and the trends in PIVKA-II level were shown in **Figure 2**. The patient was treated with the same regimen for one more cycle, and the response was evaluated as a partial response (PR) (**Figure 1**, 2 months). Then, a single session of TACE treatment was conducted, followed by a continued regimen of immunotherapy and targeted therapy. The procedure involved percutaneous puncture of the femoral artery to facilitate angiography, specifically targeting the celiac and hepatic arteries. This examination revealed a complex, tortuous hepatic artery morphology, accompanied by the presence of irregular patchy and nodular staining patterns in the right hepatic lobe, along with notable atrophy of the right hepatic artery within the portal vein. The TACE treatment was administered using a catheter, with a dose of 6 mg. Additionally, a microcatheter was strategically placed into the right hepatic artery branch to perform embolization therapy, which entailed the infusion of a mixture containing 6 ml of lipiodol and gelatin sponge particles ranging from 560 to 710μm in size. Subsequent angiography demonstrated a significant reduction in tumor staining after the embolization procedure.

**Figure 3:**
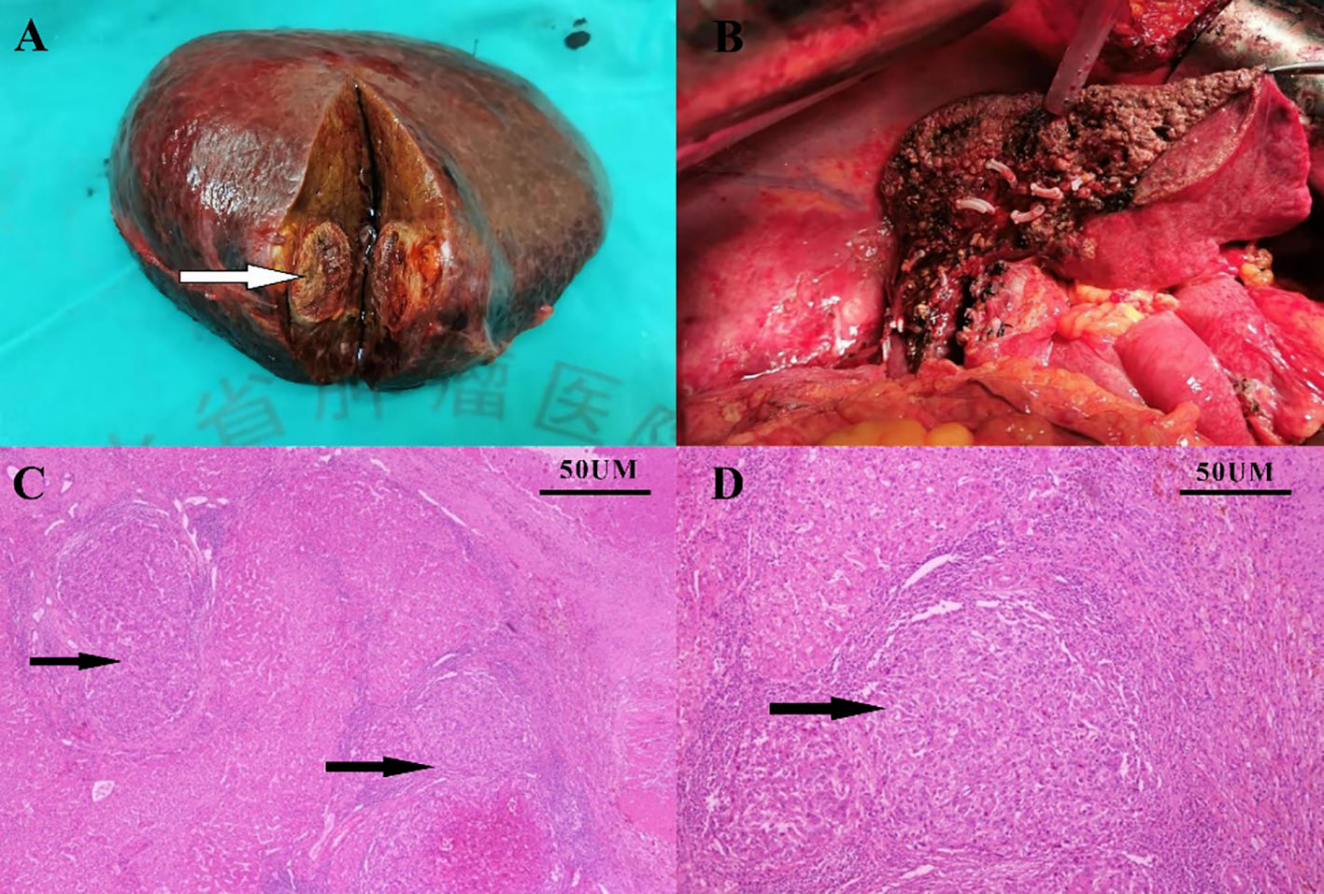
Surgical specimen and pathology. Surgically resected specimen **(A, B)**; Pathology report showed hepatocellular carcinoma **(C, D)**.

**Figure 4:**
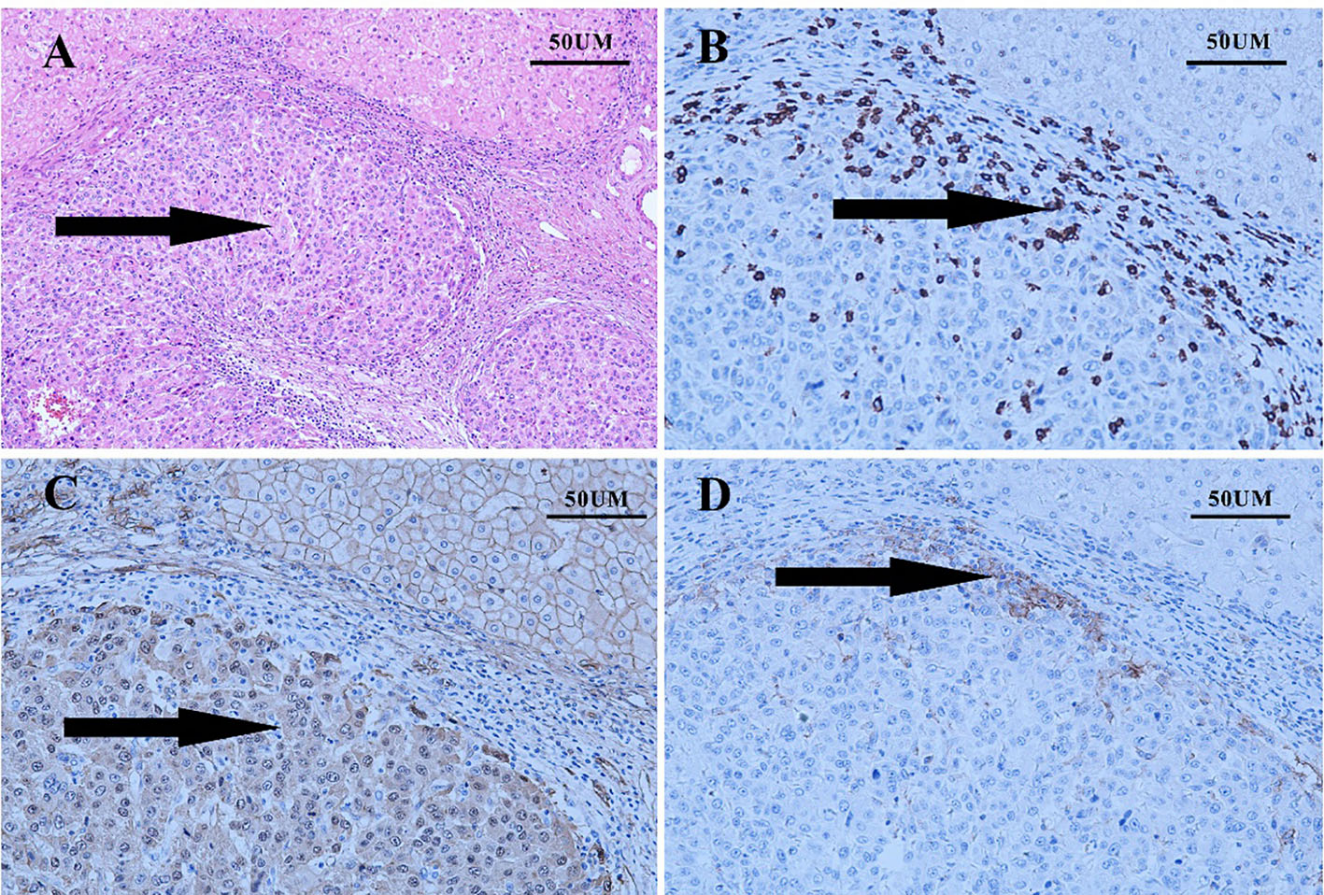
The results of supplementary immunohistochemistry experiments. **(A)** the existence of moderately differentiated hepatocellular carcinoma (Grade 2) characterized by extensive necrosis within the liver tumor and bile duct resection specimen subsequent to liver cancer treatment **(B)** the presence of CD8 (partial lymphocytes+); **(C)** B-catenin (predominantly cytoplasmic^+^ in cancer cells; predominantly membranous^+^ in surrounding liver tissue); **(D)** a PD-L1 result of CPS=15.

Four weeks later, the patient was evaluated as a PR (**Figure 1**, 3 months). However, the patient experienced discomforts such as abdominal distension and pain after this treatment and was readmitted to the hospital. CT examination showed that the main portal vein was occluded by an unclear thrombus (**Figure 1**, 4 months), which was believed to be a blood clot based on an MDT discussion. Surgical removal of the thrombus was recommended, and the patient agreed to the surgery. On August 15, 2022, the patient underwent a right hepatectomy with portal vein thrombus removal and cholecystectomy under general anesthesia (**Figure 3A, B**). The patient recovered smoothly after surgery. Pathology examination revealed moderately differentiated hepatocellular carcinoma (grade 2) with a large amount of necrosis, no microvascular invasion, and satellite nodules in the surrounding liver with fatty degeneration. The gallbladder showed chronic inflammation, and the main portal vein thrombus contained a large number of red blood cells and a few lymphocytes. Immunohistochemistry showed Glypican-3 (-), Hepatocyte (+), CK7 (-), CK19 (-), CD34 (vascular ^+^), and Ki67 (clone: SP6) (Li: 20%) (**Figure 3C, D**). Supplementary immunohistochemistry experiments were performed, yielding further pathological insights. These findings confirm the existence of moderately differentiated hepatocellular carcinoma (Grade 2) characterized by extensive necrosis within the liver tumor and bile duct resection specimen after liver cancer treatment. Immunohistochemical analysis demonstrates the presence of CD8 (partial lymphocytes+), and B-catenin (predominantly cytoplasmic+ in cancer cells; predominantly membranous+ in surrounding liver tissue), with a PD-L1 result of CPS=15 (**Figure 4**). The patient was treated with the original regimen for six months after surgery and has been without recurrence for over 34 months (**Figure 1**, 34 months). Throughout treatment, the patient predominantly experienced adverse reactions characterized by abdominal distension and general fatigue. Nevertheless, these symptoms ameliorated following appropriate symptomatic intervention.

## Discussion

Hepatocellular carcinoma combined with portal vein tumor thrombus generally has a poor prognosis. Traditional targeted drugs such as sorafenib have limited efficacy in such patients. However, this type of patient has achieved good results with immune combination targeted therapy, which also indicates the important role of immune checkpoint inhibitors combined with lenvatinib in the treatment of advanced liver cancer.

After using PD-1 immune checkpoint inhibitors and targeted therapy, the patient’s abdominal distension and diet significantly improved, which fully verified its therapeutic effect. After the second cycle of TACE, the tumor and tumor thrombus significantly reduced, abnormal prothrombin decreased, and the tumor partially relieved (PR) after evaluation. The nature of the portal vein obstruction is unclear, and a second MDT discussion was held. Based on imaging examination and abnormal prothrombin changes, the conclusion was that there was a high probability of large vessel thrombosis. After fully evaluating the patient’s physical condition, we believe that there is an opportunity for surgical resection of the tumor and removal of the thrombus. We recommend surgical treatment using the liver hilum cross-clamping method to remove the right lobe of the liver and the thrombus. The postoperative situation also confirmed the presence of thrombosis, and the patient’s survival without tumor exceeded 34 months, highlighting the importance of MDT and the need to pay attention to the treatment window period and timely surgical intervention.

Currently, PD-1 is the mainstream of immunotherapy. Its mechanism of action inhibits tumor cells from suppressing the immune system, restarts the body’s immune system, and enhances the anti-tumor effects of T cells. The activation and blockade of T cell-related important immune checkpoints are achieved through activating antibodies and blocking antibodies. Tumor cells evade the killing of T cells mainly by expressing CTLA-4 and PD-1. This approach is increasingly considered an essential part of the combined treatment for advanced liver cancer (21, 22). In our case, we used immunotherapy during the operation. We believe that the immune system needs time to restart in the treatment of liver cancer. However, targeted drugs work faster. From the observation of the patients currently being treated, especially those with liver cancer and bone metastasis with pain, clinical symptoms can only be significantly improved generally after one week of use. Numerous studies have also shown that anti-angiogenic KTI combined with immunotherapy can change the intrinsic microenvironment of tumor cells. Anti-angiogenic therapy can downregulate the expression of VEGF, inhibit tumor angiogenesis, and increase the effectiveness of treatment. Compared with sorafenib, atezolizumab plus bevacizumab has better overall survival and progression-free survival (23, 24). Shigeta et al. used an HCC *in situ* transplantation model or induced mouse model to reveal the mechanism of the combined treatment of immunotherapy and molecular targeting. In HCC, the dual blockade of PD-1 and VEGFR-2 not only promotes vascular normalization but also enhances anti-tumor immune responses (25). It was reported that 65 cases of HCC patients treated with combined immune checkpoint inhibitors and lenvatinib showed a significant improvement in OS and PFS, and there was no statistically significant difference in the incidence of adverse reactions (26). In my opinion, for liver cancer combined with portal vein tumor thrombus, accurate staging before surgery and the use of immune checkpoint inhibitors and molecular targeting drugs recommended by the guidelines can enhance the effectiveness of treatment. In the clinical application process, this treatment plan is effective for most patients. Y-90 radioembolization combined with immunotherapy is safe and effective, with an objective effective rate of 31% (26). TACE treatment for liver cancer can reduce the stage of liver cancer and may provide an opportunity for surgical resection (27). The KEYNOTE 524 study is an Ib clinical trial that evaluates the efficacy and safety of lenvatinib plus pembrolizumab as first-line treatment for advanced unresectable liver cancer (UHC). A total of 104 patients were enrolled in the study, and the ORR (confirmed remission) of patients receiving lenvatinib plus pembrolizumab treatment reached 46%, and the DCR reached 88%. The median OS of patients receiving lenvatinib plus pembrolizumab reached 22 months. In addition, regarding the postoperative pathology of liver cancer, we found significant differences in the composition of thrombi and cancer emboli. Thrombi contained more red blood cells and fewer lymphocytes, while treated cancer emboli exhibited weak activity and were accompanied by a large amount of necrotic material and infiltrated by a large number of lymphocytes. This also indicates that combined drug therapy with TACE can significantly control tumor growth.

In our observed cases, no adverse reactions of grade III or above were observed. Symptoms of hypertension were found in some cases, but they were improved after symptomatic antihypertensive treatment. A study showed that the incidence of adverse reactions in immunotherapy combined with targeted drugs did not result in treatment-related deaths. The incidence of grade III or above hypertension and palmar-plantar erythrodysesthesia syndrome (PPES) in the combination therapy group was 20% and 10.8%, respectively, compared to 17.8% and 4.4% in the single-drug group (25).

Finally, for postoperative adjuvant maintenance therapy, it is generally recommended to intervene with high-risk factors for early recurrence after liver cancer surgery with the presence of microvascular invasion (MVI+) (28–30). This patient stopped receiving postoperative adjuvant treatment after six months and achieved very good treatment results, which indicates the effectiveness of comprehensive treatment for liver cancer, especially by grasping the treatment window period. At present, there is a lack of real-time research data on the use of adjuvant therapy in HCC, and we look forward to better data being released.

## Data availability statement

The original contributions presented in the study are included in the article/supplementary material. Further inquiries can be directed to the corresponding authors.

## Ethics statement

The studies involving humans were approved by Hubei Cancer Hospital, Tongji Medical College, Huazhong University of Science and Technology. The studies were conducted in accordance with the local legislation and institutional requirements. The participants provided their written informed consent to participate in this study. Written informed consent was obtained from the individual(s) for the publication of any potentially identifiable images or data included in this article.

## Author contributions

SL, RX, CD, JT, and TY were involved in the clinical care of the patient and planned the case report. SL, SD, and TY wrote the manuscript. All authors reviewed and revised the manuscript and approved the final manuscript as submitted.
